# Case series and literature review of primary hyperoxaluria type 1 in Chinese patients

**DOI:** 10.1007/s00240-023-01494-8

**Published:** 2023-10-24

**Authors:** Jiayu Wu, Jing Song, Yanzhao He, Cheng Zhong, Qin Yang, Qiu Li, Mo Wang

**Affiliations:** 1 Department of Nephrology, Ministry of Education Key Laboratory of Child Development and Disorders, National Clinical Research Center for Child Health and Disorders, China International Science and Technology Cooperation Base of Child Development and Critical Disorders, Children’s Hospital of Chongqing Medical University, Chongqing, 400014 China; 2 University of Leeds, Woodhouse, Leeds, LS2 9JT UK

**Keywords:** Primary hyperoxaluria type 1, AGXT, Gene, China, Children

## Abstract

Based on the single-center case reports and all reported patients with primary hyperoxaluria type 1 (PH1) in China, this study discussed the clinical and genetic characteristics of this disease retrospectively. We reported and validated a novel genetic variation c.302 T > G: the clinical phenotypes of the two siblings were similar, in which both had onset in infancy, mainly manifested as renal insufficiency, and died within 6 months out of end-stage renal disease. The literature review is the first to summarize the Chinese patients with PH1 up to now. Forty-eight Chinese patients were included, containing 7 adults and 41 children. The median onset age was 51 months, and the ratio of male to female was 2.69:1. It showed a poor prognosis: 51.1% of Chinese primary hyperoxaluria type 1 patients suffered from end-stage renal disease, and 38.9% of patients died. Urolithiasis was the most common clinical manifestation both in adults and children, while infant-onset patients generally presented with renal insufficiency and had a higher mortality of 75.0%. One hundred and forty-nine AGXT mutant alleles are currently known in the Chinese population, c.33dupC and c.815_816insGA were the most common AGXT genes, accounting for 12.0% and 10.1% of allele frequencies, respectively. The exons 1, 2, 6, and 8 were the most common locations of gene variants, accounting for 78% of all variants, which will be promising targets of DNA sequencing for primary hyperoxaluria type 1.

## Introduction

Primary hyperoxalurias (PH), an autosomal recessive genetic disease with excessive endogenous oxalate production, can be divided into three types according to different enzyme defects: primary hyperoxalurias type 1 (PH1), primary hyperoxalurias type 2 (PH2), and primary hyperoxalurias type 3 (PH3). In Europe, the prevalence of PH is about 1–3 persons per million people [1, 2], while it is more common in Kuwait, Tunisia, and other countries where consanguineous marriage is common.

There are few reports about PH2 and PH3 progressing to end-stage renal disease. PH1 is the most common and severe type, and previous studies have reported that PH1 accounts for about 80% of PH, and more than 50% of PH1 patients will eventually reach end-stage renal disease (ESRD) [3, 4]. PH1 is caused by the lack or functional defect of alanine–glyoxylate aminotransferase (AGT), which is encoded by AGXT gene mutation. The clinical manifestations of PH1 are variable, ranging from early renal insufficiency in infancy to occasional urinary calculi in adults, the most common of which are recurrent urinary calculi, renal calcinosis, and progressive renal function damage. This diversity of clinical phenotypes leads to delayed or missed diagnosis in some cases. It was reported that about 20%–50% of PH1 patients entered ESRD at the time of diagnosis [4–7], and most pediatric patients who started in infancy have reached ESRD at the time of diagnosis and died within 1 year [8, 9]. The prognosis of PH1 is poor, and the current treatment methods are quite limited. Given that the diversity of the relationship between genotype and phenotype varies in ethnic groups, this study summarizes the clinical characteristics and genetic features of PH1 cases in China to promote the acknowledgment of PH1 and improve the prognosis of this disease.

## Materials and methods

### Ethical compliance and data collection

Clinical data were collected from four patients diagnosed with PH1 at the Children's Hospital of Chongqing Medical University from August 2009 to July 2021. Clinical data included basic information, clinical symptoms, signs, family history, whether the parents were close relatives, history, laboratory and imaging examinations, gene test results, treatment methods, and outcome. This study was reviewed and approved by the Ethics Committee of the Children's Hospital of Chongqing Medical University. With informed consent from the guardians of patients, we collected the peripheral blood of the four patients and their parents.

### Whole-exome sequencing and Sanger sequencing

The genomic DNA was extracted from the peripheral blood with the QIAamp DNA Mini Kit according to the instructions for DNA fragments with sizes ranging from 350 to 450 bp, and those including the adapter sequences were selected for the DNA libraries with Nanodrop 2000.

The mixture containing 1 μg of the DNA library and the GenCap probes (MyGenostics) and butter, with denaturation and annealing, then hybridized at 65 °C for 22 h. After fixation, the mixture was washed with buffer, performed 15—cycles of PCRs to amplify DNA libraries, and then purified with SPRI beads (Beckman Coulter, Brea, CA, USA). Finally, the enriched libraries were sequenced on an Illumina HiSeq 2000 sequencer (Illumina, San Diego, CA, USA).

Removed the connectors and low-quality variations (quality value ≤ 20) in the sequencing data and redundant data in the PCR process, Short Oligonucleotide Analysis Package aligner software (SOAP2.21) was used to compare with the human reference genome (UCSC hg19) to determine single nucleotide polymorphisms (SNPs). The genome analysis toolkit software 3.7 was used to detect deletions and insertions. Finally, check the short read length comparison through MagicViewer, and confirm the candidate SNPs, deletions, and insertions.

Genomic DNA from all available family members was obtained for Sanger sequencing. The PCR samples were visualized on agarose gels, purified, and sequenced on an ABI PRISM 3730 genetic analyzer (Applied Biosystems; Thermo Fisher Scientific, Inc.) using the terminator cycle sequencing method. Sites of variation were identified by comparing DNA sequences with the corresponding GenBank (www.ncbi.nlm.nih.gov) reference sequences.

### Pathogenicity analysis

We analyzed the five algorithms to evaluate the pathogenicity of non‑synonymous variants: PolyPhen (http://genetics.bwh.harvard.edu/pph2/), Provean (http://provean.jcvi.org/index.php), MUTATION TASTER(http://www.mutationtaster.org/), Combined Annotation Dependent Depletion (https://cadd.gs.washington.edu/snv) and Mendelian Clinically Applicable Pathogenicity(http://bejerano.stanford.edu/MCAP/). And we also predicted the secondary and spatial structures of the novel variant (http://www.sbg.bio.ic.ac.uk/phyre2/).

Conservation analysis was performed through multiple sequence alignment of representative species with GenBank (http://www.ncbi.nlm.nih.gov).

### Literature review

"Primary hyperoxygenas type 1", "AGXT", and "China or Chinese" were used as keywords or free words to search in PubMed. We also searched VIP (http://www.cqvip.com/), and CNKI (https://www.cnki.net/), Wanfang (http://www.wanfa ngdata.com.cn/index.html) with the same keywords in Chinese. All literature from the establishment of the database to March 1, 2022, was searched to screen Chinese PH1 patients. Exclusion criteria: no gene test results; If the case is repeated, select the clearest and the most detailed literature; The patient is a Mestizo of China and other countries.

## Results

### Case series

#### Patient 1

The proband was the first child of his Chinese parents, with average birth weight and length. There was no abnormality of pregnancy and delivery. Since the age of 4 months, the patient was noticed to have slow growth and development and could not sit alone or point fingers at nine months old. At 7 months old, he exhibited edema, oliguria, and abnormal renal function. Physical exam suggested that his height was 65 cm (< 3rd percentile), and weighted 7.1 kg (< 3rd percentile), with moderate non-pitting edema of bilateral lower limbs and perineum. On admission, the routine blood hemoglobin was 81 g/L, serum urea was 33.49 mmol/L, and serum creatinine was 844.6umol/L in another hospital. Accompanied by severe metabolic acidosis, there was also hypertension and proteinuria. Detailed laboratory examinations are shown in Table 1. Renal ultrasound showed enhanced echo of both kidney parenchyma and fluid accumulation in both kidney calyx areas. The patient was diagnosed with end-stage renal disease and accepted multiple hemodialysis and kidney-saving treatments, however, it was disappointing that he had repeated swelling and oliguria without frequent hemodialysis. The patient finally died from uremia at the age of 10 months old.

**Table 1:** Clinical features and auxiliary examinations of 4 PH1 patients in China

Patient	P1	P2	P3	P4
Gender	M	F	M	M
Age of onset	7 months	4 months	4 months	23 months
Initial visit	9 months	5 months	5 months	23 months
Onset symptoms	Fever, cough, and convulsion	Diarrhea, vomit	Cough, diarrhea, and convulsion	Urethral stones
Family history	+	+	−	−
Growth retardation	+	−	−	−
Proteinuria	+	−	+ / −	−
Hematuria	−	−	+	−
Scr (15.4–90.4 umol/L)	844.6	454	616.6	118
BUN (2.42–6.72 mmol/L)	33.49	24.45	14.53	5.4
eGFR ml/(min.1.73m2)	3.7	6.8	5.2	91
Blood calcium (2.23–2.8 mmol/L)	2.4	2.17	1.9	2.43
Blood phosphorus (1.3–2.1 mmol/L)	4.2	2.93	2.87	1.68
PTH (10-69 pg/ml)	62.3	188	UA	68.9
Nephrocalcinosis	+	+	+	+
Urinary tract stones	−	−	−	+
Prognosis	Die	Die	Die	Follow up
Mutations	c.302 T > G (p.L101R) c.653C > T(p.S218L)	c.302 T > G(p.L101R) c.653C > T(p.S218L)	c.26delC(p.T9fs) c.32C > G (p.P11R)	c.33dupC(p.K12Qfs*) c.33dupC(p.K12Qfs*)

Note: *P* patient; *M* male, *F* female, *Scr* serum creatinine, *BUN* blood urea nitrogen, *eGFR* estimated glomerular filtration rate, *PTH* parathyroid hormone, *UA* unavailable

#### Patient 2

The patient was the second child of her parents and also the sibling sister of patient 1. Her height and weight were similar to her peers, and no significant evidence of growth retardation had been observed. The patient was referred to our department at 5 months of age. Suffering from repeated diarrhea and vomiting, the patient gradually appeared edema and oliguria. Physical exams showed edema of bilateral eyelids and lower extremities. There was anemia of the lowest hemoglobin 68 g/L, metabolic acidosis, and hypertension, without proteinuria. Based on similar clinical manifestations and the Sanger sequencing results, the diagnosis of PH1 is definite. Owing to the potentially poor prognosis, her parents refused renal replacement therapy and gave up. The patient died shortly after discharge with uremia.

#### Patient 3

The third patient had a standard growth rate and steady cognitive development which was similar to peers. The patient presented to our department with convulsions and persistent anuria at 5 months of age, although had been accepted to hemodialysis in another hospital. He had significantly elevated serum creatinine 803 umol/L and serum urea nitrogen 43.4 mmol/L. Laboratory examination revealed moderate anemia of hemoglobin 86 g/L and severe coagulation dysfunction with activated partial prothrombin time > 240 s, detailed data ware shown in Table 1. The patient had been accepted component blood transfusion, repeated blood purification, and other symptomatic and supportive treatment, but the renal function continued to deteriorate. At the age of 7 months, the patient died of renal failure.

#### Patient 4

**Fig. 1:**
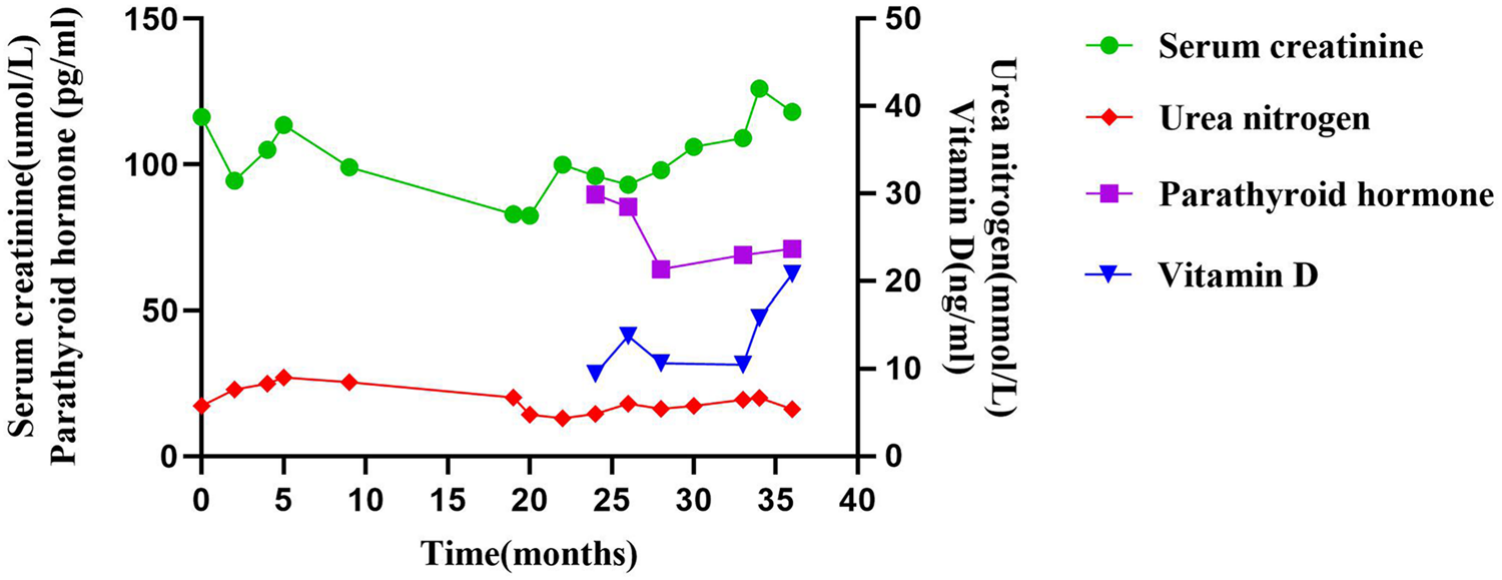
Serum creatinine, urea nitrogen, parathyroid hormone and vitamin D levels of patient 4 during follow-up

The fourth patient was born prematurely in a Chinese family, whose growth and development are similar to those of peers. At 23 months old, he was admitted to our hospital due to lumbago and vomiting for the first time and experienced the surgery of endoscopic urethral stone removal. Since then, the patient has undergone multiple surgeries due to recurrent urinary tract stones, including bladder and ureterectomy lithotripsy, ureteroscopic lithotripsy, kidney stone laser lithotripsy, and percutaneous nephrostomy of the left kidney. Between 2009 and 2015, he had the acute onset of urinary lithiasis five times. After that, the patient missed follow-ups for 3 years and started regular follow-up in our outpatient department since the 6th onset of urinary lithiasis (about 2 years ago). Over the 2 year-follow-up periods, there was no hematuria, proteinuria, anemia, hypertension, or metabolic acidosis. Although the renal function showed a tendency of gradual deterioration, the proband was in the chronic kidney disease stage 2 (Fig. 1). Eleven years after onset, he was detected with an elevated parathyroid hormone of 89.7 pg/ml (10–69 pg/ml) and a decrease in vitamin D levels, with standardised value of blood calcium and phosphorus, while his estimated glomerular filtration rate (eGFR) was 105 ml/min 1.73 m^−2^. The patient was given the therapy of calcitriol and retested normal after 4 months. Recent ultrasound results suggest left renal atrophy and multiple stones in both kidneys, and the last eGFR was 91 ml/min 1.73 m^−2^.

All clinical findings and laboratory examinations of four patients are shown in Table 1.

### Genetic sequencing and pathogenicity analysis

**Fig. 2:**
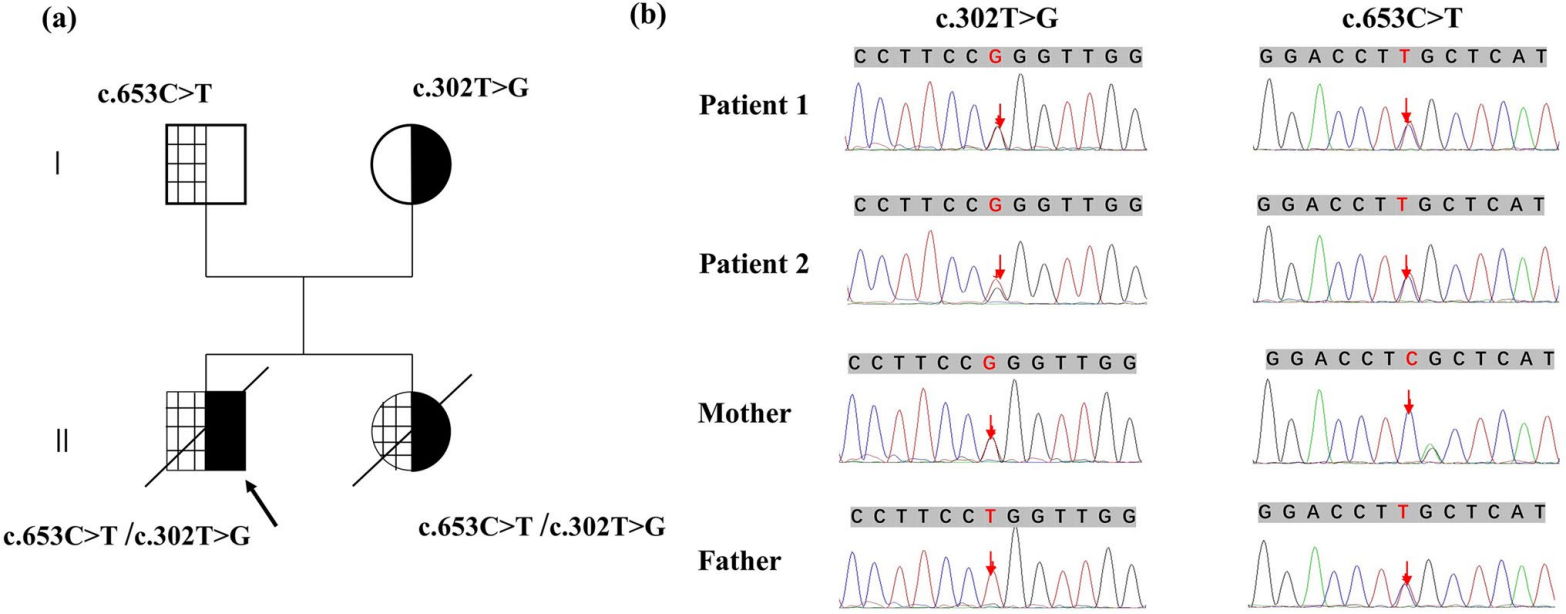
Family diagram and variants of patients 1 and 2. (**a**) The family diagram of patient 1 and patient 2, and patient 1 was the proband (The black arrow). (**b**) Sanger sequencing results of patient 1 and patient 2, show the mutations of c.302 T > G and c.653C > T (The red arrow)

**Fig. 3:**
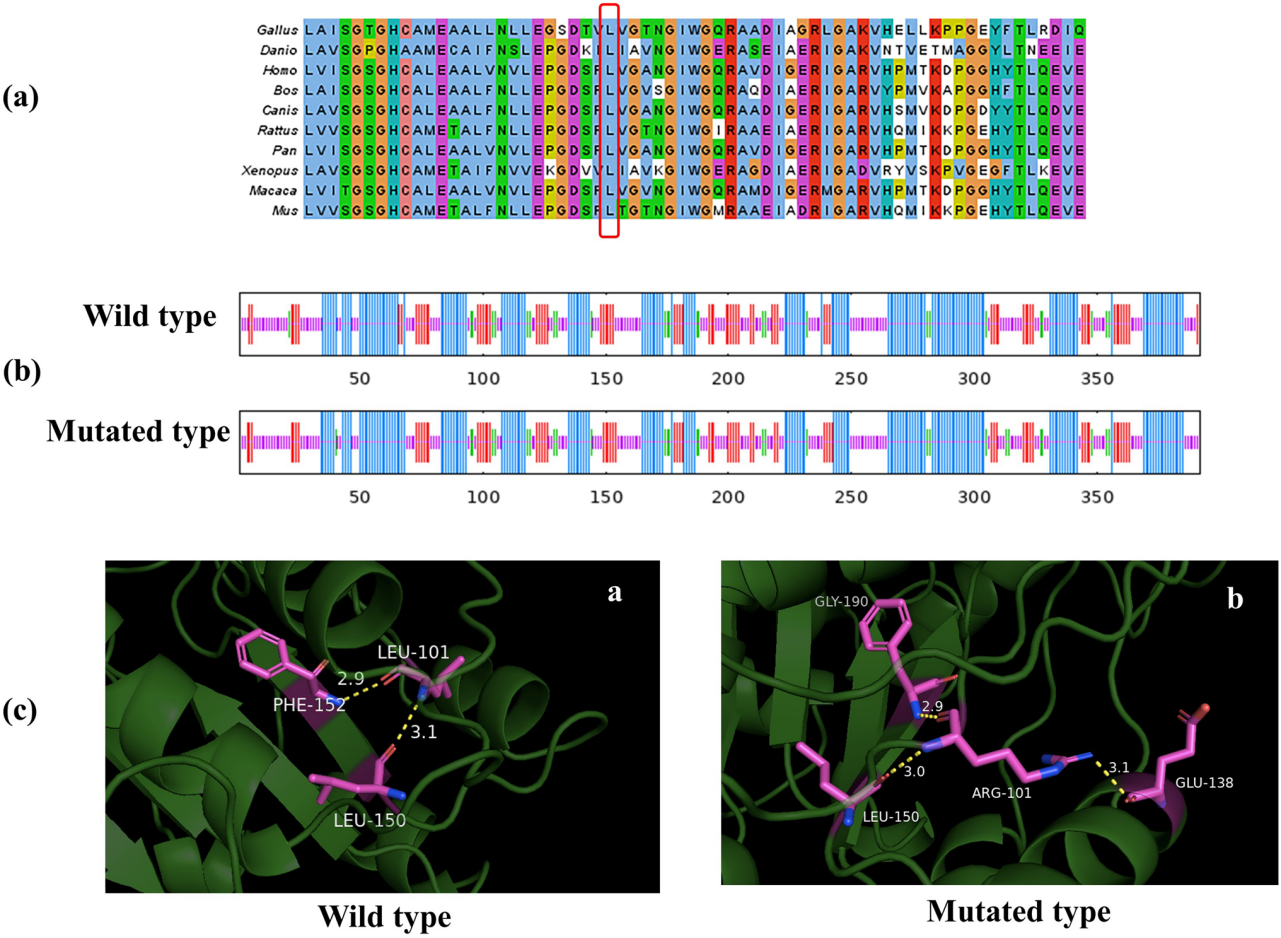
Pathogenicity analysis of novel missense variant: c.302 T > G (p.L101R). (**a**) Conservation analysis: the red box shows the changed amino acid of mutation. The variant is located at the conserved leucine residues and highly conserved in ten species. (**b**) Secondary structure: alpha helix (blue), extended strand (red), beta-turn (green), and random coil (purple). (**c**) Spatial structure: yellow dashed lines represent hydrogen bonds. The predicted wild-type structure shows that the amino acid residues interact with PHE-152 and LEU-150, respectively; There is no longer a hydrogen bonding force between the mutant protein and PHE-152, instead of GLU-138 and GLY-190

Gene sequencing results show that patient 1 and patient 2 carried AGXT gene compound heterozygous mutations, c.302 T > G (p.L101R) and c.653C > T (p.S218L), which were missense mutations inherited from their parents (Fig. 2). The novel variation c.302 T > G was first reported. Thus, we performed the following pathogenicity: (1) Conservation analysis: the mutated amino acid changes were located at the highly conserved leucine residues of representative species (Fig. 3a); (2) Bioinformatics protein function prediction software: PROVEAN, PolyPhen − 2, M-CAP, CADD, MUTATION TASTER are predicted to be pathogenic (Table 2). Through the predicted spatial structure, it was found that the hydrogen bonding forces between amino acid residues changed after mutation, which may affect the function of the protein. Moreover, through the search of the ClinVar database and human gene mutation database, the pathogenic variation of the same mutation site c.302 T > C (p.L101P) can be retrieved [10, 11]. According to American Medical Genetics and Genomics (ACMG) standards and guidelines, c.302 T > G is considered to be possibly pathogenic.

**Table 2:** The pathogenicity analysis of the variant c.302 T > G mutation

Nucleotide	PROVEAN	PolyPhen − 2	M-CAP	CADD	MUTATION TASTER
c.302 T > G	−5.478 Pathogenic	0.954 Possible pathogenic	0.758 Possibly pathogenic	26.9 Pathogenic	Disease-causing

Another variant c.653C > T(p.S218L), located near pyridoxal phosphate cofactor binding sites 201–221, has been reported, it may destroy AGT-PLP binding [12, 13]. Two AGXT-related gene mutations, including frameshift mutation c.26delC(p.T9fs) and missense mutation c.32C > G(p.P11R) were found in patient 3, have been reported to be pathogenic [14]. And sequencing results revealed the homozygous mutation c.33dupC of the AGXT gene in patient 4, which was a frameshift mutation from his parents and also the mutation of foreign hot spots [15, 16].

### Literature review

**Fig. 4:**
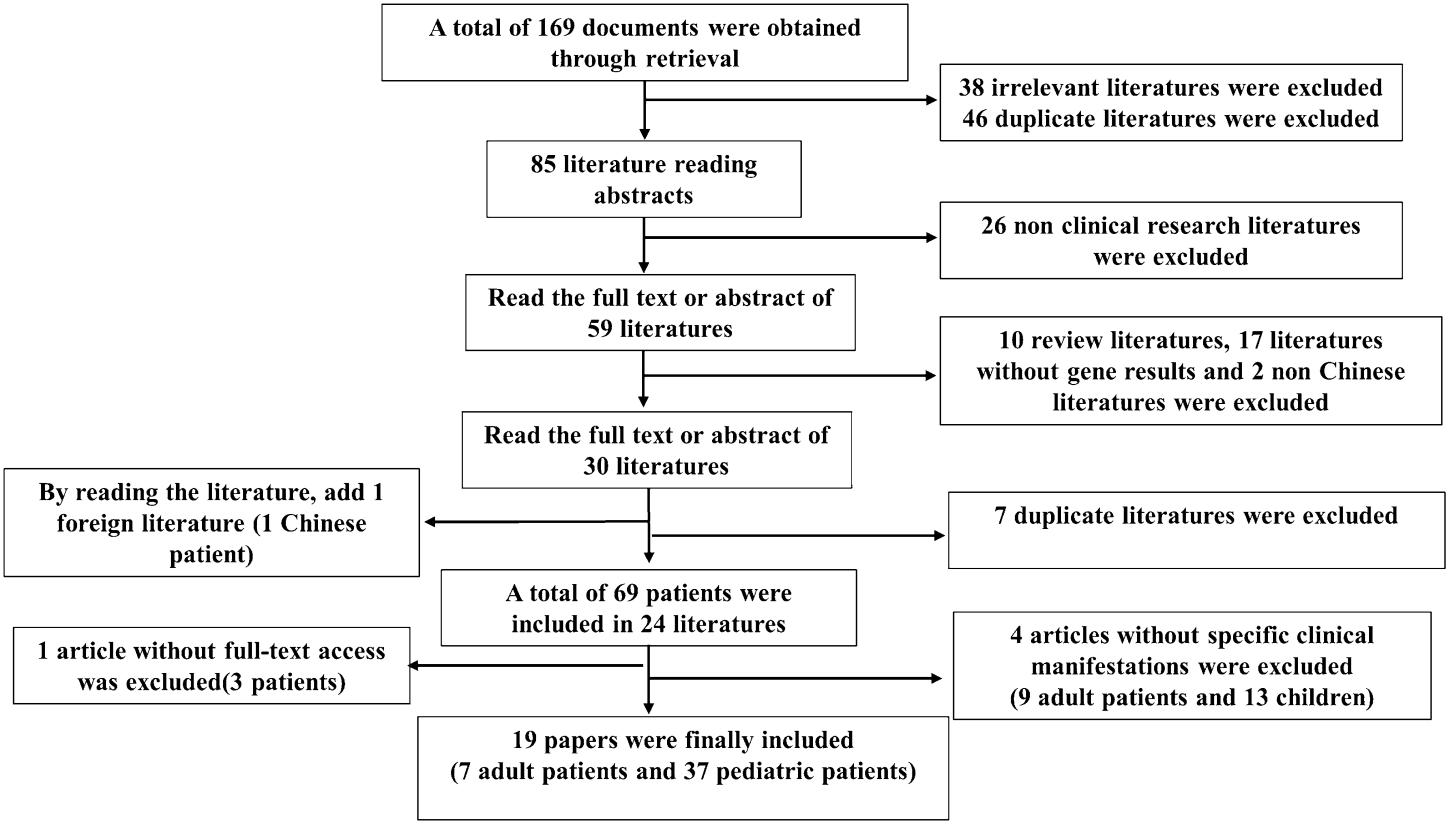
The flow chart of the literature review

For the first time, we reviewed the literature of all PH1 patients in China [13, 14, 17–38]. A total of 73 cases were analyzed, including four cases in this study, from the establishment of various databases until March 1, 2022 (Fig. 4). Four patients had a mutation c.145A > C(p.M49L) in addition to two AGXT alleles [27, 37], and Yuen et al.'s literature failed to obtain the full text [18]. Therefore, 149 AGXT mutant alleles are currently known in the Chinese population (Tables 3 and 4). For Chinese patients, c.33dupC and c.815_816insGA were the most common mutations, accounting for 12.0% and 10.1% of allele frequencies, respectively, and then c.32C > G and c.679_680del. Exons 1, 2, 6, and 8 were also the most common locations in the gene, accounting for 78% of all variants. In terms of variant types, missense mutations were the most common, followed by frameshift mutations.

**Table 3:** AGXT gene variants of PH1 patients in China

Exon	Frequency	Nucleotide changes	Amino acid changes	Variant type	Single gene frequency
Exon 1	44/149 (29.6%)	c.2 T > C	p.M1T	Missense	5/149 (3.4%)
		c.25_26 insC	p.T9Tfs	Frameshift	1/149 (0.7%)
		c.26delC	p.T9fs	Frameshift	1/149 (0.7%)
		c.26_27insC	p.T9fs	Frameshift	2/149 (1.3%)
		c.28_29delCCinsA	p.P10Tfs	Frameshift	1/149 (0.7%)
		c.32C > G	p.P11R	Missense	9/149 (6.0%)
		c.33dupC	p.K12Qfs	Frameshift	18/149 (12.0%)
		c.97C > A	p. Leu33Met	Missense	1/149 (0.7%)
		c.107G > A	p. Arg36His	Missense	1/149 (0.7%)
		c.121G > T	p. Gly41Trp	Missense	1/149 (0.7%)
		c.145A > C	p.M49L	Missense	4/149 (2.7%)
Exon 2	20/149 (13.3%)	c.190A > T	p.I64F	Missense	2/149 (1.3%)
		c.215A > T	p.N72I	Missense	7/149 (4.7%)
		c.242C > A	p.Ser81X	Nonsense	2/149 (1.3%)
		c.302 T > G	p.L101R	Missense	2/149 (1.3%)
		c.331C > T	p. Arg111*	Nonsense	1/149 (0.7%)
		c.332G > A	p.Arg111Gln	Missense	2/149 (1.3%)
		c.346G > A	p.Gly116Arg	Missense	4/149 (2.7%)
Exon 3	2/149 (1.3%)	c.364C > T	p. Arg122*	Nonsense	2/149 (1.3%)
Exon 4	5/149 (3.3%)	c.466G > A	p.G156R	Missense	1/149 (0.7%)
		c.473C > T	p.S158L	Missense	2/149 (1.3%)
		c.517 T > c	p.Cys173Arg	Missense	2/149 (1.3%)
Exon 5	7/149 (4.6%)	c.551C > A	p.Ser184	−	1/149 (0.7%)
		c.557C > T	p.Ala186Val	Missense	2/149 (1.3%)
		c.577delC	p.Leu193Phefs*19	Frameshift	2/149 (1.3%)
		c.590G > A	p.Arg197Gln	Missense	2/149 (1.3%)
Exon 6	27/149 (18.2%)	c.605 T > A	p.I202N	Missense	5/149 (3.4%)
		c.614C > T	p.S205L	Missense	4/149 (2.7%)
		c.638C > T	p.A213V	Missense	1/149 (0.7%)
		c.653C > T	p.S218L	Missense	2/149 (1.3%)
		c.667A > c	p.Ser223Arg	Missense	2/149 (1.3%)
		c.672G > T	p.Lys209Asn	Missense	1/149 (0.7%)
		c.679_680del	p.K228Efs	Frameshift	7/149 (4.7%)
		679-(IVS6 + 2)delAAgt	−	Splice	5/149 (3.4%)
Exon 7	1/149 (0.7%)	c.740 T > G	p.Leu247Arg	Missense	1/149 (0.7%)
Exon 8	25/149 (16.9%)	c.815_816insGA	p.L272fs	Frameshift	15/149 (10.1%)
		c817insAG	p.S275Rfs	Frameshift	1/149 (0.7%)
		c.823_824dupAG	p.Ser275Argfs*38	Frameshift	4/149 (2.7%)
		c.824G > A	p.S275N	Missense	1/149 (0.7%)
		c.824_825insAG	p.S275Rfs	Frameshift	2/149 (1.3%)
		c.829_830insA	p.Ala277fs	Frameshift	1/149 (0.7%)
		c844C > T	p.Gln282*	Nonsense	1/149 (0.7%)
Exon 9	2/149 (1.4%)	c.864G > A	p.Trp288X	Nonsense	1/149 (0.7%)
		c.909delG	p.Q303fs	Frameshift	1/149 (0.7%)
Exon 10	6/149 (4.0%)	c.997A > T	p.R333*	Nonsense	3/149 (2.0%)
		c.1015delG	p.V339Sfs*2	Frameshift	1/149 (0.7%)
		c.1049G > A	p. Gly350Asp	Missense	2/149 (1.3%)
Exon 11	10/149 (6.7%)	c.1072–2A > G	−	Splice	1/149 (0.7%)
		c. 1079G > A	p. Arg360Gln	Missense	6/149 (4.0%)
		c.1161C > A	p.C387X	Nonsense	3/149 (2.0%)

**Table 4:** The distribution and outcomes of the most common variants of AGXT in Chinese PH1 patients

Parameters	Frequency (*n*,%)
The most common variants	Number of variants (*n* = 149)
c.33dupC	18/149 (12.0)
c.815_816insGA	15/149 (10.1)
c.32C > G	9/149 (6.0)
c.679_680del	7/149 (4.7)
c.215A > T	7/149 (4.7)
Mutation type	Total number of alleles^a^ (*n* = 148)
Missense	72/148 (48.6)
Frameshift	57/148 (38.5)
Nonsense	13/148 (8.8)
Splice	6/148 (4.1)
Gene function category	Total number of PH1 patients^b^ (*n* = 47)
Truncation/truncation	19/47 (40.4)
Missense/missense	14/47 (29.8)
Truncation/ missense	11/47 (23.4)
Truncation/splice	2/47 (4.3)
Missense/ splice	1/47 (2.1)
Gene function category	Patients with ESRD/PH1 patients with renal function outcome
Truncation/truncation	7/19 (36.8)
Missense/missense	9/14 (64.3)
Truncation/missense	6/10 (60.0)
Truncation/splice	1/2 (50.0)
Missense/splice	1/1 (100.0)
Gene function category	Dead patients/patients with the outcome
Missense/missense	4/12 (33.3)
Truncation/ missense	3/8 (37.5)
Truncation/truncation	2/12 (16.7)
Truncation/splice	1/1 (100.0)
Missense/splice	0/1 (0.0)
Exon location of the variant gene	The proportion of patients with ESRD^c^
Exon 1	15/32 (46.9)
Exon 2	10/12 (83.3)
Exon 6	6/13 (46.2)
Exon 8	10/19 (52.6)
Exon location of the variant gene	The proportion of patients who died^d^
Exon 1	7/24 (29.2)
Exon 2	4/11 (36.4)
Exon 6	5/11 (45.5)
Exon 8	1/12 (8.3)

Note: Truncation mutations: nonsense and frameshift mutations; a: exclude a patient with unknown amino acid mutation results; b: Total number of PH1 patients with genotype and their corresponding clinical manifestations included; c: PH1 patients without renal function outcomes were excluded; d: PH1 patients with no outcome were excluded; Renal function outcome: end-stage renal disease, renal insufficiency or normal; outcome: survive or die

We finally included 48 Chinese patients with follow-up results in this study (Table 5), 7 adults and 41 children, and males constituted the majority of 72.9%, the median onset age of Chinese patients was 51 months. The clinical manifestations were mainly divided into two types: urolithiasis and renal insufficiency, and the former was more common (41/48, 85.4%). Children with PH1 accounted for the majority in the literature review (77.1%), and the age of onset was generally earlier, as the median onset age of pediatric patients was 38 months. 51.1% of Chinese PH1 patients developing ESRD, and 38.9% of PH1 patients died.

**Table 5:** Clinical manifestation and outcomes of PH1 patients in different countries

	Chinese patients of PH1	Total pediatric patients	Pediatric patients		Adult patients	Mandrile et al., 2014 [7]	M'Dimegh et al., 2017 [39]	Hopp et al., 2015 [16]	Abid et al., 2023 [40]	Wannous et al., 2023 [41]
			< 1 year old	≥ 1 year old						
Gender (male/female)	35/13	30/11	7/2	22/10	5/2	300/226	94/52	NA	141/64	20/20
The median age of onset	51 months	38 months	4 months	55 months	28 years old	NA	NA	5.2 years old	Under 5 years old	3 years old
Region	China					Europe	Tunisia	America	South Asia	Syria
Main clinical manifestations (*n*,%)										
Urolithiasis	41/48, 85.4	34/41, 82.9	2/9, 22.2	32/32,100.0	7/7,100.0	316/526, 60.1	74/146, 51.0	49/160, 30.6	41.3%	28/40, 70.0
Renal insufficiency	7/48, 14.5	7/41, 17.1	7/9, 77.8	0/32, 0.0	0/7, 0.0	NA	NA	NA	67.2%	29/40, 72.5
Outcomes^a^ (*n*, %)										
ESRD	24/47, 51.1	18/40, 45.0	7/8, 87.5	11/31, 35.5	6/7, 85.7	267/458, 58.0	89/146, 61.0	NA	50%	NA
Death^b^	14/36, 38.9	8/29, 27.6	6/8, 75.0	1/20, 5.0	3/6, 50.0	67/477, 14.0	NA	NA	NA	13/28, 46.4

Note: a: According to Fig. 4, 48 Chinese PH1 patients had specific gene results and corresponding clinical manifestations, and one patient who lost to follow-up was excluded. b: PH1 patients with no outcomes were excluded. NA: Unavailable

## Discussion

Alanine glyoxylate aminotransferase (AGT) is a homodimeric protein encoded by the AGXT gene, with pyridoxal phosphate (PLP) as its coenzyme, and exists typically in liver peroxisomes specifically, which catalyzes the conversion of glyoxylate to glycine [42]. Deficiency or functional defect of AGT will lead to the accumulation of glyoxylate in the peroxisome, which is then diffused into the cytoplasm of hepatocytes and oxidized to oxalate by lactate dehydrogenase (LDH). Oxalate is mainly insoluble calcium oxalate, which is mostly excreted by the kidneys. When calcium oxalate is supersaturated in the urine for a long time, it is deposited in the kidneys in the form of stones, resulting in repeated urinary tract stones, nephrocalcinosis, and progressive kidney damage.

The clinical manifestations of PH1 patients range from asymptomatic to isolated or recurrent nephrolithiasis, nephrocalcinosis, and renal damage. The heterogeneity of manifestations increases the difficulty of diagnosis. Clinical characteristics revealed that PH1 patients have a poor prognosis: 51.1% of Chinese PH1 patients developing ESRD, and 38.9% of PH1 patients died. We observed that children with onset after 1 year of age and adult patients both have urinary calculi as the main manifestation, while whose onset within 1 year of age generally present with renal insufficiency, poor prognosis, and high mortality (6/8, 75.0%).

The incidence rate and severity of PH1 vary in different regions and ethnics. In a large European registry of 526 PH1 patients [7], urolithiasis or nephrocalcinosis was the most frequent symptom, and the similar proportion of ESRD, whereas the mortality rate of patients with ESRD was lower due to the active treatment in the later stage, such as liver and kidney transplantation. Another large cohort study [16] of 247 American PH1 patients showed that the renal survival of patients at 20, 40, and 60 years of age were 76%, 43%, and 12%, respectively; moreover, it was found that although the gene profiles of mutations were partially different between African Americans and European Americans, the incidence predictions were similar. Another study of 205 patients with PH1 from South Asia [40] revealed that the morbidity of nephrocalcinosis and ESRD were similar to Chinese. While for the Syrians, whose median age of onset was 3 years, 72.5% had renal insufficiency at the initial visit and the mortality rate of PH1 was as high as 46.4%, which may be related to the local special social environment and medical level. For African countries such as Libya, Tunisia, and Morocco, the incidence of PH1 is higher owing to consanguineous marriage. A study involving 146 Tunisian PH1 patients showed that male patients (64.4%) and patients under 20 years of age (60.3%) were the majority. 56.8% of patients have positive close relatives, and 61% developed ESRD [39]. A retrospective study of 53 PH1 pediatric patients in Libya also revealed that urolithiasis or nephrocalcinosis was the most common manifestation [43]. Therefore, we found that PH1 is a male-dominated disease and is common in children in China, mainly manifested as urolithiasis. The rates of ESRD are roughly similar across regions, but the mortality rates vary in regions, which we think is related to the level of care available in different regions. PH1 patients have a poor prognosis, and current treatment options are limited, early confirmation of the diagnosis of PH1 and appropriate treatment is critical.

The AGXT gene is located on 2p37.3, with more than 200 AGXT mutations have been reported (http://www.hgmd.cf.ac.uk). Most Chinese patients are sporadic cases, and there is no PH1-related epidemiological data in China. For the first time, we performed a detailed summary and analysis of clinical manifestations and genetic results in Chinese PH1 patients. 149 AGXT mutant alleles are currently known in the Chinese population, c.33dupC and c.815_816insGA were the most common in the Chinese population, accounting for 12.0% and 10.1% of allele frequencies (Tables 3 and 4), respectively, and then c.32C > G, c.679_680del, c.215A > T. We searched the gnomAD and found two gene frequencies of the above common variations in Asian populations: c.32C > G (0.001407), c.215A > T (0.0001088). In South Asia, p.Gly350Asp, p.Leu101Pro, p.Gly190Arg, Cys173_His174delinsTrpAsn, and c.33dupC account for 61% of the mutant alleles [40], which were partially different from Chinese patients.

p.F152I, p.G170R, p.I244T, and c.33dupC were common mutations in Europe PH1 patients, located in AGXT exons 1, 4, and 7, accounting for more than 50% of alleles; And p.G170R was found in more than 50% of American PH1 families. While p.R289H is the most common allele in African Americans, accounting for about 33% of the mutated alleles, p.G170R is common and relatively unique in European Americans [16]. However, the above common mutations p.F152I, p.G170R, and p.I244T have not been found in the Chinese or South Asian population. For African countries, Tunisian data demonstrates that genetic variation mainly includes p.I244T (43.4%), p.Gly190Arg (21.4%), and c.33dupC (13.1%) [39], and p.I244T is the most common in Libyan children [43]. As a consequence, genetic variant differences may be related to race and region. There is an exception, c.33dupC accounts for about 11%–15% of the allele frequency in foreign reports [15, 16, 39], which is consistent with Asian and African, whose regional differences are not obvious.

Targeted sequence analysis of AGXT exons 1, 4, and 7 have been proposed for first-line testing in the UK. The sensitivity for detection of a single mutation in the biopsy-proven population is 75%, and for two pathogenic variants is 50% [44]. While in the Arab population, 70% of PH1 patients have gene variants located in exons 1, 2, 5, 7, and 10. DNA sequencing of the above five exons showed a diagnostic sensitivity of 82.22% [43, 45–47]. In the Chinese population, gene variants in exons 1, 2, 6, and 8 are the most common, accounting for 78% of all variants. Moreover, we found that the proportion of patients who achieved ESRD or death was higher in these common exons. The proportion of patients with ESRD in exons 2 and 8 was 83.3% and 52.6%, respectively, and the proportion of patients with death in exon 6 was 45.5%, possibly associated with poor prognosis, especially exon 2. These exons are both hot spots of mutation in the Chinese population and may be associated with poor prognosis. Thus, they are worth screening as targets for DNA sequencing.

For frameshift mutation c.33dupC, a stop codon can be formed, so that the amino acid can terminate the translation in advance, forming a truncated protein and resulting in the complete loss of AGT protein expression and enzymatic activity [22]. Therefore, PH1 patients with this mutation tend to have a more severe phenotype, and the homozygous state for this mutation occurs mainly in pediatric cases and is rarely reported in late-onset cases [48–50]. Mbarek IB et al. reported eight children with PH1, all homozygous for the c.33dupC mutation, 75% died of the disease at a median age of 2.5 years, and other patients also had severe urolithiasis, all of which had developed ESRD [51]. Murad, H et al. reported 12 pediatric patients with PH1 homozygous for the c.33dupC mutation, 10 of whom suffered from renal failure or died. Patient 4 in this study is a homozygote for this mutation, mainly manifested as recurrent urinary tract stones, whose duration of disease has lasted for over 12 years. Although renal function has declined, it is still in the first stage of chronic kidney disease. The phenotype appears much milder than most other children with variants at this locus. Most adult patients are characterized by urolithiasis, and the progression of renal function damage is slower than that of children. There are reports of ESRD reaching the age of 70. The above heterogeneity of genotype and phenotype supports the idea that other enzymes may be involved in oxalate synthesis, or that glyoxylate metabolism may be regulated through interactions with modifying genes and/or environmental factors [48, 51]. However, in general, patients with urolithiasis as the main presentation were significantly younger than other mutation types when they carried frameshift mutations [22].

The limitations of this study are: Chinese patients are mostly sporadic cases, these included patients need longer follow-up, and whether AGXT exons 1, 2, 6, and 8 are associated with poor prognosis still needs to be further verified with larger samples; For the genotype and phenotype of PH1 patients, the heterogeneity between them needs to be further explored at the molecular level.

## Conclusion

This study reported and validated a novel genetic variation c.302 T > G, which mainly manifested as renal insufficiency, and died of ESRD within 6 months. This study also summarized all Chinese PH1 patients for the first time, which is characterized by male-dominated disease and common in children in China, usually with poor prognosis, especially in patients less than 1 year of age. In Chinese patients, exons 1, 2, 6, and 8 of AGXT were the common locations of variants, c.33dupC and c.815_816insGA were common mutation sites, which are expectant to be targets for DNA sequencing.

## Data Availability

The authors confirm that the data supporting the findings of this study are available within the article.
